# Exercise and obesity in fibromyalgia: beneficial roles of IGF-1 and resistin?

**DOI:** 10.1186/ar4187

**Published:** 2013-02-27

**Authors:** Jan L Bjersing, Malin Erlandsson, Maria I Bokarewa, Kaisa Mannerkorpi

**Affiliations:** 1 Department of Rheumatology and Inflammation Research, Institute of Medicine, Sahlgrenska Academy, University of Gothenburg, Guldhedsgatan 10, Box 480, 40530 Göteborg, Sweden; 2 Sahlgrenska University Hospital, Rheumatology. Gröna stråket 14. 413 45 Göteborg. Sweden; 3 Sahlgrenska University Hospital, Physiotherapy and Occupational therapy. Vita stråket 13. 413 45 Göteborg. Sweden; 4 University of Gothenburg Centre for Person-centered Care (GPCC), Sahlgrenska Academy, Gothenburg, Sweden

## Abstract

**Introduction:**

Severe fatigue is a major health problem in fibromyalgia (FM). Obesity is common in FM, but the influence of adipokines and growth factors is not clear. The aim was to examine effects of exercise on fatigue, in lean, overweight and obese FM patients.

**Methods:**

In a longitudinal study, 48 FM patients (median 52 years) exercised for 15 weeks. Nine patients were lean (body mass index, BMI 18.5 to 24.9), 26 overweight (BMI 25 to 29.9) and 13 obese. Fatigue was rated on a 0 to 100 mm scale (fibromyalgia impact questionnaire [FIQ] fatigue) and multidimensional fatigue inventory (MFI-20) general fatigue (MFIGF). Higher levels in FIQ fatigue and MFIGF indicate greater degree of fatigue. Free and total IGF-1, neuropeptides, adipokines were determined in serum and cerebrospinal fluid (CSF).

**Results:**

Baseline FIQ fatigue correlated negatively with serum leptin (r = -0.345; *P *= 0.016) and nerve growth factor (NGF; r = -0.412; *P *= 0.037). In lean patients, baseline MFIGF associated negatively with serum resistin (r = -0.694; *P *= 0.038). FIQ Fatigue associated negatively with CSF resistin (r = -0.365; *P *= 0.073). Similarly, FIQ fatigue (r = -0.444; *P *= 0.026) and MFIGF correlated negatively with CSF adiponectin (r = -0.508; *P *= 0.01). In lean patients, FIQ fatigue (P = 0.046) decreased after 15 weeks. After 30 weeks, MFIGF decreased significantly in lean (MFIGF: *P *= 0.017), overweight (MFIGF: *P *= 0.001), and obese patients (MFIGF: *P *= 0.016). After 15 weeks, total IGF-1 increased in lean (*P *= 0.043) patients. ∆Total IGF-1 differed significantly between lean and obese patients (*P *= 0.010). ∆Total IGF-1 related negatively with ∆MFIGF after 15 weeks (r = -0.329; *P *= 0.050). After 30 weeks, ∆FIQ fatigue negatively correlated with ∆NGF (r = -0.463; *P *= 0.034) and positively with ∆neuropeptide Y (NPY) (r = 0.469; *P *= 0.032). Resistin increased after 30 weeks (*P *= 0.034). ∆MFIGF correlated negatively with ∆resistin (r = -0.346; *P *= 0.031), being strongest in obese patients (r = -0.815; *P *= 0.007). In obese patients, ∆FIQ fatigue after 30 weeks correlated negatively with ∆free IGF-1 (r = -0.711; *P *= 0.032).

**Conclusions:**

Exercise reduced fatigue in all FM patients, this effect was achieved earlier in lean patients. Baseline levels of resistin in both serum and CSF associated negatively with fatigue. Resistin was increased after the exercise period which correlated with decreased fatigue. Changes in IGF-1 indicate similar long-term effects in obese patients. This study shows reduced fatigue after moderate exercise in FM and indicates the involvement of IGF-1 and resistin in these beneficial effects.

**Trial registration:**

ClinicalTrials.gov: NCT00643006

## Introduction

Severe fatigue, together with pain, is a major health problem in fibromyalgia (FM) [1,2] and is considered to be equally important to pain [3] in causing impaired work ability and restricted social participation [4]. It is associated with depression, sleep quality and pain [5]. Obesity is common in FM, with a reported prevalence between 40 and 70% [6-8]. Increased body mass index (BMI) generally correlates with increased levels of pain and fatigue in FM [7,9-11]. In chronic fatigue syndrome, symptom severity is suggested to be associated with metabolic syndrome [12]. Weight levels may affect neuroendocrine regulation of pain and fatigue through several pathways.

There is evidence for deregulation of the growth hormone/insulin-like growth factor (IGF-1) signaling in obesity [13,14] and an inverse relationship between total IGF-1 levels and BMI has been reported [15,16]. In FM patients we have recently reported a beneficial role of IGF-1 and exercise with regard to pain [17]. These results were in line with previous findings indicating that IGF-1 has a protective role in FM [18,19] and that IGF-1 promotes resilience to stress and pain in the central nervous system (CNS) [20,21]. Furthermore, growth hormone deficiency is shown to be associated with fatigue and reduced cognitive speed [22].

Recently, several factors, termed adipokines, which are produced in adipose tissue, have been found to have important regulatory roles in both inflammation and nutrition. Adiponectin is one of these adipokines and was initially isolated in adipocytes. Adiponectin regulates energy balance both in peripheral tissues and via the CNS [23,24]. Adiponectin receptors are distributed widely in the brain, affecting appetite, metabolism and autonomic function [25,26]. Adiponectin is negatively correlated with depression [27,28] and has antidepressant-like effects in both lean and diet-induced obese mice [29].

Resistin is considered to be an adipokine with unusual properties, and a potential link between inflammation and metabolic disease [30]. It is expressed in human macrophages and has documented regulatory effects on metabolism, adipogenesis and inflammatory reactions [31-33]. Peripheral levels of resistin are upregulated in subjects with insulin resistance and in obesity [34,35], and resistin signaling involves both toll-like receptor (TLR)4 [36] and the IGF-1 receptor [37].

Leptin is another important adipokine. It is a major product of adipose tissue, is increased in obesity and is a central regulator of satiety and body weight [38,39], as well as reproduction, mood and emotion [40-42]. Induction of satiety is mediated by leptin receptors in hypothalamic neuropeptide Y (NPY), producing neurons [43-46]. Leptin has anxiolytic effects in mice [47,48] and is involved in allodynia in a neuropathic pain model [49]. NPY is an abundant neuropeptide, both in the peripheral and in the central nervous system. NPY is an important modulator of hippocampal and thalamic circuits, with the potential to affect a number of different functions in the brain. It is also involved in neuroprotection, neurogenesis and neuroinflammation [50]. NPY is altered in FM patients, possibly involving the hypothalamic-pituitary-adrenal axis [51-54]. NPY is also altered in chronic fatigue syndrome [55,56] and during stress and depression [57]. Disturbed neuropeptide levels with elevated substance P (SP) [58-60] and nerve growth factor (NGF) [61] have previously been found in cerebrospinal fluid in FM. Recent evidence also implicate glial activation in FM with increased IL-6 and IL-8 in cerebrospinal fluid [62].

The aim of the study was to examine the long-term effects of aerobic exercise on fatigue, in lean, overweight, and obese women with FM. Changes in serum free bioactive IGF-1, total IGF-1, IGF binding protein (IGFBP)3, adipokines and neuropeptides were studied to gain a better understanding of the biological mechanisms involved in fatigue in FM.

## Materials and methods

### Study design

This study is a part of a previously reported randomized controlled exercise study, studying the effects of a moderate-to-high-intensity Nordic walking (NW) program and a supervised low-intensity walking (LIW) program. The effects of Nordic walking on body function were reported previously [63], showing that NW resulted in better improvement in the 6-minute walk test (6MWT) and aerobic capacity, when compared with LIW.

### Subjects

The criteria for inclusion were as follows: women with FM, aged 20 to 60 years, with interest in exercising outdoors twice a week for 15 weeks, who agreed to undergo blood tests at baseline and after the exercise period. To ensure that the patients would manage the planned aerobic exercise, they were required to complete a bicycle test at 50 watts to fulfill the inclusion criteria. All included patients managed to perform the test. They were also invited to participate in an examination of cerebrospinal fluid; however, this was not a criterion for inclusion. FM was defined by the American College of Rheumatology (ACR) 1990 criteria [64]: a history of long-lasting generalized pain and pain in at least 11 of 18 tender points examined by manual palpation.

The criteria for exclusion were as follows: patients who could not speak or read Swedish; presence of other severe somatic or psychiatric disease; BMI <18.5; ongoing or planned physical therapy, including exercise, and inability to attend at the times of the planned exercise sessions.

Forty-nine patients, 26 of them undertaking NW and 23 undertaking LIW, had blood tests at baseline, after 15 weeks of exercise, and at 30 weeks of follow-up, as described in the previous report [17]. One of the patients had BMI <18.5 and was therefore excluded from this study. In total, 48 patients with FM formed the study population.

The median age of patients was 52 (48 to 56, interquartile range) years and their median duration of symptoms was 11 (7 to 15) years. The median number of tender points was 15 (13 to 16). Eighty-two percent of patients were taking analgesics during the study and 63% were taking antidepressants or sedatives. Nine patients were lean (BMI 18.5 to 24.9), 26 patients were overweight (BMI 25.0 to 29.9) and 13 were obese (BMI ≥30.0). After separating the patients into BMI groups we found a similar distribution in the NW and LIW group. In the lean group, four subjects participated in NW and five in LIW. In the overweight group fifteen participated in NW and eleven in LIW. In the obese group seven participated in NW and six in LIW.

### Exercise intervention

The patients were randomized to either the moderate-to-high-intensity NW program (n = 26) or the supervised LIW program (n = 22). Both supervised aerobic exercise programs were conducted twice a week for 40 to 45 minutes for 15 weeks. Patients had blood tests before, after 15 weeks, and after 30 weeks. Pain and fatigue did not significantly change in any of the exercise groups after 15 weeks, while scores in the Multidimensional Fatigue Inventory (MFI-20) [66] subscale of General Fatigue (MFIGF) improved in both groups after 30 weeks. As no differences in fatigue or pain were found between the two exercise groups, and BMI was similarly distributed in both exercise groups, the analyses in this study were conducted on the total population (n = 48), irrespective of exercise intensity. Compliance was assessed as attendance at exercise sessions. It was slightly higher among the lean group, whose attendance was 71%, while it was 64% in the overweight group. Attendance in the obese group was 57%.

### Clinical measurements

Fatigue was rated on a visual analog scale (0 to 100) of the Fibromyalgia Impact Questionnaire (FIQ) [65] which gives an estimation of global fatigue, as well as with the MFIGF [66], which estimates fatigue by questions related to feeling fit, tired and rested. Both instruments reflect fatigue during the last week, and a higher score indicates more severe fatigue.

### Blood and cerebrospinal fluid (CSF) sampling

Serum was collected at rest (n = 48) at baseline, after 15 weeks in the exercise program, and at 30 weeks of follow-up (n = 41). Serum samples were acquired by venipuncture of the cubital vein. Twenty-six patients agreed to participate in an examination of cerebrospinal fluid (CSF) at baseline. CSF was collected through lumbar puncture through the lumbar vertebrae (L)3/L4 interspace. Collected blood and CSF samples were centrifuged at 800 g for 3 minutes, aliquoted, and stored frozen at -70°C until use.

### Laboratory analyses

Samples were analyzed with enzyme-linked immunosorbent assay (ELISA) using commercially available kits. Assays specific for human adiponectin (DY1065, 62.5 pg/ml), human leptin (DY 398. 31 pg/ml), human resistin (DY1359, 31 pg/ml), free bioactive IGF-1 (DY291, 4 pg/ml) and IGFBP3 (DY675, 0.125ng/ml) were purchased from R and D Systems (Minneapolis, MN, USA). Serum total IGF1 was measured by solid-phase, enzyme-labeled chemoluminescent immunoassay (Immulite 2000 IGF1, L2KGF2) on an Immulite 2000 (Siemens Medical Solutions Diagnostics, Los Angeles, CA, USA). An assay specific for NPY (FEK-049-03, 1 pg/ml) was purchased from Phoenix Pharmaceuticals (Burlingame, CA, USA). The human NGF-specific assay was purchased from Promega (Madison, WI, USA; 4 pg/ml). All assays were run according to recommendations of the manufacturers. Ordinary colorimetric ELISA was read with a Spectramax 340 from Molecular Devices (Sunnyvale, CA, USA), and fluorescent ELISA assays were read with a Mithras LB940 from Berthold Technologies (Bad Wildbad, Germany).

### Statistics

Descriptive data are presented as median and interquartile range. The Wilcoxon signed-rank test was used for comparisons of continuous variables within groups. Baseline data and differences in changes in lean patients were compared by the Mann-Whitney *U*-test with overweight and obese patients, respectively. Relationships between the variables were examined with the Spearman correlation coefficient. To control for possible Type I errors, the upper limit of the number of false significant results was calculated by the following formula:

$$\left(\text{Number of tests}-\text{Number of significant tests on level of alpha}\right)\;*\;\text{Alpha}/\left(\text{1-Alpha}\right).$$

### Ethics

The study was approved by the ethics committee of Sahlgrenska University Hospital. Written and verbal information was given to all patients, and written consent was obtained from all patients.

## Results

### Relationship between obesity, fatigue, adipokines and IGF-1

Several differences in fatigue and adipokines were found in relation to obesity. Patients with normal BMI (18.5 to 24.9) had higher baseline fatigue (97 mm) compared to overweight patients with BMI 25 to 29.9 (74 mm; *P *= 0.008), while no significant differences were found compared to obese patients with BMI ≥30 (88 mm; *P*-value not significant.) (Table 1). Baseline levels of adiponectin were higher in lean compared to overweight patients (*P *= 0.013) and compared to obese patients (*P *= 0.003) (Table 2). Leptin levels were lowest in lean patients and tended to be higher in overweight patients (*P *= 0.067) and were highest in obese patients (*P *<0.001). Resistin levels did not differ significantly between groups. Total IGF-1 was higher in lean patients compared to overweight patients (160.0 vs 113.0 ng/ml, *P *= 0.026) and obese patients (160.0 vs 106.5, *P *= 0.056), (Table 3). Serum free IGF-1 and IGFB3 did not differ between the groups.

**Table 1:** Clinical data in lean, overweight and obese patients with fibromyalgia

	Lean (group 1)		Overweight (group 2)		Obese (group 3)		Lean vs overweight	Lean vs obese
	
	Median (range)	n	Median (range)	n	Median (range)	n	*P*-value^a^	
Age, years	52.0 (33.5 to 54.0)	9	53.0 (48.0 to 56.0)	26	51.0 (47.0 to 55.5)	13	0.288	0.647
BMI, kg/m^2^	23.5 (21.5 to 24.0)	9	28.1 (27.1 to 29.5)	26	32.7 (30.8 to 35.7)	13	<0.001	<0.001
Tender points, n	14.0 (12.5 to 15.5)	9	15.0 (13.0 to 17.0)	26	15.0 (13.0 to 16.0)	13	0.224	0.393
Baseline FIQ fatigue, mm	97.0 (77.5 to 98.0)	9	74.0 (52.8 to 88.5)	26	88.0 (72.5 to 91.5)	13	0.008	0.110
Baseline MFIGF	20.0 (17.0 to 20.0)	9	16.5 (13.0 to 20.0)	26	17.0 (14.5 to 20.0)	13	0.061	0.209
Change in FIQ fatigue after 15 weeks, mm	-7.0 (-13.5 to 0.0) *P *= 0.046	8	-0.5 (-16.3 to 8.3) *P *= 0.485	26	-8.0 (-20.5 to 2.0) *P *= 0.059	13	0.368	0.972
Change in FIQ fatigue after 30 weeks, mm	-4.5 (-12.8 to 0.3) *P *= 0.161	8	-2.0 (-7.0 to 8.8) P = 0.966	24	-2.5 (-6.8 to 4.5) *P *= 0.345	12	0.254	0.473
Change in MFIGF after 15 weeks	-2.0 (-4.2 to 0.0) *P *= 0.084	8	0.0 (-1.2 to 1.0) *P *= 0.515	26	0.0 (-3.5 to 1.0) *P *= 0.641	13	0.164	0.336
Change in MFIGF after 30 weeks	-3.0 (-5.5 to -2.0) *P *= 0.017	8	-2.0 (-3.0 to 0.0) *P *= 0.001	24	-3.0 (-4.0 to -1.0) *P *= 0.016	12	0.147	0.624

Levels of fatigue (FIQ and MFIGF) at baseline (0 weeks), during training (15 weeks) and after training (30 weeks). Lean patients had BMI 18.5 to 24.9; overweight patients had BMI 25.0 to 29.9. Obese patients had BMI ≥30.0. Median values and interquartile range are indicated. ^a^Mann-Whitney *U*-test. n, number; BMI, body mass index; FIQ, Fibromyalgia Impact Questionnaire; MFIGF, Multidimensional Fatigue Inventory subscale of General Fatigue.

**Table 2:** Adipokines in lean, overweight and obese patients

	Lean (group 1)			Overweight (group 2)			Obese (group 3)			Comparison of groups			
	
	Baseline	∆15 wks	∆30 wks	Baseline	∆15 wks	∆30 wks	Baseline	∆15 wks	∆30 wks	Groups	At baseline	Change after 15 weeks	Change after 30 weeks
	Median (range)	Median (range) *P*-value^a^	Median (range) *P*-value^a^	Median (range)	Median (range) *P*-value^a^	Median (range) *P*-value^a^	Median (range)	Median (range) *P*-value^a^	Median (range) *P*-value^a^		*P*-value^b^	*P*-value^b^	*P*-value^b^
Adipo-nectin ng/ml	18620.8 (5707.0 to 23038.0)	783.3 (-3501.6 to 4270.7)	488.5 (-1047.7 to 6069.1)	4580.4 (2939.7 to 9634.0)	400.0 (-438.0 to 1681.0)	578.0 (-1764.5 to 1281.3)	4745.5 (2864.9 to 5879.0)	1011.3 (-382.6 to 3273.8)	1206.9 (-1157.4 to 3811.5)	Lean vs overweight	0.013	0.591	0.346
	n = 9	*P *= 0.441 n = 9	*P *= 0.398 n = 7	n = 26	*P *= 0.122 n = 25	*P *= 0.861 n = 25	n = 13	*P *= 0.133 n = 13	*P *= 0.214 n = 9	Lean vs obese	0.003	0.794	1.000
Leptin ng/ml	16012.4 (10250.4 to 25627.3)	2136.2 (-4133.4 to 5400.4)	-4455.8 (-10473.7 to 2250.7)	27658.2 (19222.8 to 45504.1)	1417.0 (-6055.4 to 6858.3)	1760 (-9912.1 to 9778.1)	45300.0 (34456.5 to 86307.4)	5269.7 (-5534.8 to 9055.5)	1446.4 (-3059.4 to 8129.1)	Lean vs overweight	0.067	0.939	0.161
	n = 9	*P *= 0.515 n = 9	*P *= 0.128 n = 7	n = 26	*P *= 0.778 n = 25	*P *= 0.443 n = 25	n = 13	*P *= 0.422 n = 13	*P *= 0.594 n = 9	Lean vs obese	<0.001	0.471	0.142
NPY pg/ml	114.5 (86.6 to 624.6)	19.0 (-10.7 to 98.5)	6.7	122.8 (80.5 to 164.9)	-7.7 (-27.4 to 31.3)	9.0 (-8.2 to 27.3)	113.7 (76.0 to 553.8)	21.8 (1.3 to 225.0)	44.4 (18.3 to 1272.7)	Lean vs overweight	0.82	0.178	0.824
	n = 4	*P *= 0.465 n = 4	*P *= 0.18 n = 2	n = 16	*P *= 0.959 n = 16	*P *= 0.256 n = 15	n = 6	*P *= 0.075 n = 6	*P *= 0.043 n = 5	Lean vs obese	0.914	0.762	0.095
Resistin pg/ml	14768.2 (12312.3 to 27191.5)	927.7 (-3165.5 to 3897.5)	1006.1 (-8555.9 to 4448.6)	14419.1 (13786.6 to 16531.2)	437.3 (-1698.2 to 2634.9)	701.1 (-573.9 to 3872.6)	14951.6 (12671.2 to 20016.0)	1189.2 (-1110.2 to 2292.4)	3110.7 (-534.1 to 4311.0)	Lean vs overweight	0.67	1.000	0.789
	n = 9	*P *= 0.678 n = 9	*P *= 1.000 n = 7	n = 26	*P *= 0.465 n = 23	*P *= 0.051 n = 25	n = 13	*P *= 0.221 n = 13	*P *= 0.214 n = 9	Lean vs obese	0.794	0.845	0.837

Serum levels of adiponectin, leptin, NPY and resistin at baseline (0 weeks), change (∆) during training (15 weeks) and change after training (30 weeks). Median values and interquartile range are indicated. ^a^Wilcoxon signed rank test. ^b^Mann-Whitney *U*-test. NPY, neuropeptide Y.

**Table 3:** Serum free IGF-1, total IGF-1 and IGFB3 in lean, overweight and obese patients

	Lean (group 1)			Overweight (group 2)			Obese (group 3)			Comparison of groups			
	
	Baseline Median (range)	∆15 wks Median (range) *P*-value^a^	∆30 wks Median (range) *P*-value^a^	Baseline Median (range)	∆15 wks Median (range) *P*-value^a^	∆30 wks Median (range) *P*-value^a^	Baseline Median (range)	∆15 wks Median (range) *P*-value^a^	∆30 wks Median (range) *P*-value^a^	Groups	At baseline *P*-value^b^	Change after 15 weeks *P*-value^b^	Change after 30 weeks *P*-value^b^
Free IGF-1 ng/ml	4.3 (2.3 to 4.6)	0.8 (-1.1 to 1.4)	0.1 (-5.5 to 0.9)	3.1 (1.7 to 6.3)	-0.4 (-1.5 to 0.5)	-0.7 (-1.6 to 0.6)	5.7 (3.4 to 8.0)	-1.2 (-4.7 to 0.4)	-2.1 (-4.7 to -0.1)	Lean vs overweight	0.697	0.171	0.900
	n = 9	*P *= 0.515 n = 9	*P *= 0.753 n = 6	n = 26	*P *= 0.174 n = 26	*P *= 0.053 n = 24	n = 13	*P *= 0.173 n = 13	*P *= 0.017 n = 10	Lean vs obese	0.164	0.051	0.368
IGFB3 ng/ml	1605.6 (1179.4 to 2397.9)	-260.7 (-578.6 to 36.3)	-568.5 (-899.7 to 123.5)	1492.2 (1079.2 to 1999.4)	106.0 (-370.3 to 942.3)	349.2 (-282.8 to 767.0)	1645.2 (1420.6 to 2002.8)	-19.9 (-622.3 to 544.8)	282.0 (-869.5 to 1558.2)	Lean vs overweight	0.563	0.107	0.095
	n = 8	*P *= 0.093 n = 8	*P *= 0.249 n = 6	n = 26	*P *= 0.397 n = 25	*P *= 0.128 n = 25	n = 10	*P *= 0.878 n = 10	*P *= 0.735 n = 7	Lean vs obese	0.897	0.460	0.628
Total IGF-1 ng/ml	160.0 (123.0 to 187.0)	33.0 (0.0 to 51.0	65.0 (17.8 to 94.3)	113.0 (90.3 to 151.8	10.0 (-13.5 to 19.0)	34.0 (-0.9 to 48.0)	106.5 (95.8 to 157.8)	-1.0 (-21.8 to 4.5)	19.0 (6.0 to 29.0)	Lean vs overweight	0.026	0.065	0.125
	n = 7	*P *= 0.043 n = 7	*P *= 0.116	n = 24	*P *= 0.309 n = 17	*P *= 0.006	n = 12	*P *= 0.255 n = 12	*P *= 0.017	Lean vs obese	0.056	0.010	0.036

Levels of serum free IGF-1, total serum IGF-1 and serum IGFB3 at baseline (0 weeks), change (**∆**) during training (15 weeks) and change after training (30 weeks). Median values and interquartile range are indicated. ^a^Wilcoxon signed rank test. ^b^Mann-Whitney *U*-test. IGF-1, insulin-like growth factor-1; IGFB3, insulin-like growth factor-binding protein-3.

Fatigue was also related to adipokine levels and IGF-1 levels. Baseline fatigue was negatively correlated with serum levels of leptin (*r *= -0.345, *P *= 0.016, n = 48) and NGF (*r *= -0.412, *P *= 0.037, n = 26) (Table 4). Leptin correlated negatively with total IGF-1 (*r *= -0.354, *P *= 0.020, n = 43) and positively with NPY (*r *= 0.472, *P *= 0.015, n = 26) and NGF. Serum free IGF-1 correlated with total IGF-1 (r = 0.366; *P *= 0.016; n = 43) and IGFB3 (r = 0.361; *P *= 0.016; n = 44) and with NGF (r = 0.401; *P *= 0.042; n = 26). In lean patients, baseline fatigue was negatively associated with resistin levels (r = -0.694; *P *= 0.038; n = 9).

**Table 4:** Fatigue versus adipokines, IGF-1 and neuropeptides

		FIQ fatigue	Adiponectin	Leptin	Resistin	Free IGF-1	Total IGF-1	IGFB3	NPY	NGF
MFIGF	*r* *P* n	0.623 <0.001 48	0.107 0.468 48	-0.074 0.618 48	-0.125 0.398 48	0.024 0.871 48	0.254 0.100 43	0.039 0.801 44	0.150 0.464 26	0.141 0.493 26
FIQ Fatigue	*r* *P* n	1.000 48	0.143 0.333 48	-0.345 0.016 48	-0.111 0.451 48	-0.032 0.829 48	0.198 0.203 43	0.155 0.315 44	0.004 0.984 26	-0.412 0.037 26
Adiponectin	*r* *P* n		1.000 48	0.032 0.827 48	0.123 0.405 48	-0.200 0.172 48	0.148 0.343 43	-0.143 0.355 44	0.245 0.227 26	-0.151 0.460 26
Leptin	*r* *P* n			1.000 48	0.153 0.300 48	0.131 0.374 48	-0.354 0.020 43	0.082 0.595 44	0.472 0.015 26	0.426 0.030 26
Resistin	*r* *P* n				1.000 48	-0.103 0.486 48	0.036 0.817 43	-0.043 0.782 44	0.023 0.912 26	0.148 0.470 26
Free IGF-1	*r* *P* n					1.000 48	0.366 0.016 43	0.361 0.016 44	0.167 0.414 26	0.401 0.042 26
Total IGF-1	*r* *P* n						1.000 43	0.188 0.250 39	0.085 0.706 22	0.233 0.296 22
IGFB3	*r* *P* n							1.000 44	0.395 0.046 26	0.198 0.332 26
Neuropeptide Y (NPY)	*r* *P* n								1.000 26	0.340 0.089 26

Correlation between baseline fatigue (FIQ and MFIGF), serum free IGF-1, serum levels of total IGF-1, IGFB3, adipokines and neuropeptides. r, Spearman's correlation coefficient; *P*, *P*-value; n, number; MFIGF, Multidimensional Fatigue Inventory subscale of General Fatigue; FIQ, Fibromyalgia Impact Questionnaire; IGF, insulin-like growth factor; IGFB3, insulin-like growth factor-binding protein-3; NGF, nerve growth factor.

FIQ Fatigue was negatively associated with resistin levels in CSF (*r *= -0.365, *P *= 0.073, n = 25) (Table 5). A similar pattern was seen for CSF levels of adiponectin with negative correlations to FIQ fatigue (*r *= -0.444, *P *= 0.026, n = 25) and MFIGF (*r *= -0.508, *P *= 0.01, n = 25).

**Table 5:** Correlation between baseline fatigue and cerebrospinal fluid levels of adipokines and neuropeptides

	MFIGF	Adiponectin CSF	Leptin CSF	Resistin CSF	NPY CSF	NGF CSF
FIQ fatigue	*r *= 0.623 *P *<0.001 n = 48	*r *= -0.444 *P *= 0.026 n = 25	*r *= -0.233 *P *= 0.263 n = 25	*r *= -0.365 *P *= 0.073 n = 25	*r *= -0.111 *P *= 0.607 n = 24	*r *= -0.243 *P *= 0.243 n = 25
MFIGF		*r *= -0.508 *P *= 0.01 n = 25	*r *= -0.189 *P *= 0.365 n = 25	*r *= -0.316 *P *= 0.123 n = 25	*r *= 0.014 *P *= 0.947 n = 24	*r *= 0.219 *P *= 0.293 n = 25

Correlation between baseline fatigue (FIQ and MFIGF) and cerebrospinal fluid levels of adiponectin, leptin, resistin, NPY and NGF. *r*, Spearman's correlation coefficient; *P*, *P*-value; n, number; FIQ, Fibromyalgia Impact Questionnaire; MFIGF, Multidimensional Fatigue Inventory subscale of General Fatigue; CSF, cerebrospinal fluid; NPY, neuropeptide Y; NGF, nerve growth factor.

### Influence of exercise on fatigue

In the group as a whole, FIQ fatigue was decreased after 15 weeks (median -4, interquartile range -15 to 4, *P *= 0.024, n = 47), and after 30 weeks both FIQ fatigue (-2, -7 to 4.5; *P *= 0.252, n = 44) and MFIGF were decreased (-2, -4 to -1, *P *<0.001, n = 44). In lean patients, FIQ fatigue (-7, 13.5 to 0, *P *= 0.046) and MFIGF (-2, -4.2 to 0, *P *= 0.084) were decreased after 15 weeks (Table 1). After 30 weeks, MFIGF decreased significantly in lean patients, (-3, -5.5 to -2, *P *= 0.017), overweight patients (-2, -3 to 0, *P *= 0.001) and obese patients (-3, -4 to -1, *P *= 0.016), and the direction of change in FIQ fatigue was the same although not significant.

### Influence of exercise on levels of IGF-1, adipokines and neuropeptides

As mentioned above, total IGF-1 was highest in lean patients and lower in overweight and obese patients (Table 3). After 15 weeks, total IGF-1 was further increased in lean patients (33 ng/ml, 0 to 51, *P *= 0.043, n = 7), but was unchanged in overweight patients (10, -13.5 to 19, *P *= 0.309, n = 17) and in obese patients (-1, -22 to 4.5, *P *= 0.255). The change in total IGF-1 differed significantly between lean and obese patients (*P*= 0.010). After 30 weeks, serum free IGF-1 was significantly decreased in obese patients (-2.1 ng/ml, -4.7 to -0.1, *P *= 0.017, n = 10) and in overweight patients (-0.7, -1.6 to 0.6, *P *= 0.053, n = 24) but was unchanged in lean FM patients. Resistin increased in the group as a whole after 30 weeks (944 pg/ml, -819 to 4299, *P *= 0.034, n = 41), while adiponectin and leptin were unchanged after 30 weeks. NPY levels were increased after 30 weeks (11.1, 0.6 to 33, *P *= 0.017, n = 22), this increase was only significant in obese patients (44.4, 18.3 to 1272, *P *= 0.043, n = 5). Adiponectin levels were increased in the whole group of FM patients after 15 weeks (695, -432 to 1891, *P *= 0.022, n = 47) but not after 30 weeks.

### Changes in fatigue in relation to IGF-1, adipokines and neuropeptides

Change in MFIGF (∆MFIGF) after 15 weeks was negatively correlated with ∆total IGF-1 (*r *= -0.329, *P *= 0.050, n = 36). In lean patients, ∆total IGF-1 was correlated with ∆resistin in serum (*r *= 0.829, *P *= 0.021, n = 7) after 15 weeks; ∆free IGF-1 after 15 weeks of exercise correlated positively with ∆NGF in serum (*r *= 0.428, *P *= 0.029, n = 26).

After 30 weeks, ∆free IGF-1 was negatively correlated with ∆NPY (*r *= -0.563, *P *= 0.006) (Table 6). ∆FIQ fatigue was correlated negatively with ∆NGF (*r *= -0.463, *P *= 0.034, n = 21) and positively with ∆NPY (*r *= 0.469, *P *= 0.032, n = 21). ∆MFIGF correlated negatively with ∆resistin (*r *= -0.346, *P *= 0.031, n = 39); this negative correlation was strong in obese patients (*r *= -0.815, *P *= 0.007, n = 9) (Table 7) but was much weaker in lean and overweight patients. In obese patients, ∆FIQ fatigue after 30 weeks was negatively correlated with ∆free IGF-1 (*r *= -0.711, *P *= 0.032, n = 9) and ∆adiponectin (r = -0.753, *P *= 0.019) (Table 7).

**Table 6:** Change in fatigue versus change in adipokines, IGF-1 and neuropeptides

		∆MFIGF 30 wks	∆Adiponectin 30 wks	∆Leptin 30 wks	∆Resistin 30 wks	∆Free IGF-1 30 wks	∆Total IGF-1 30 wks	∆IGFB3 30 wks	∆NGF 30 wks	∆NPY 30 wks
∆FIQ fatigue 30 wks	*r* *P* n	0.415 0.005 44	-0.227 0.164 39	-0.111 0.502 39	-0.016 0.923 39	-0.147 0.385 37	0.106 0.549 34	0.250 0.141 36	-0.463 0.034 21	0.469 0.032 21
∆MFIGF 30 wks	*r* *P* n	1.000 44	-0.096 0.561 39	0.280 0.084 39	-0.346 0.031 39	-0.201 0.232 37	-0.075 0.672 34	0.382 0.022 36	0.043 0.852 21	0.209 0.364 21
∆Adiponectin 30 wks	*r* *P* n		1.000 41	-0.016 0.922 41	0.011 0.943 41	0.002 0.989 39	0.091 0.600 36	-0.241 0.145 38	0.097 0.669 22	-0.361 0.099 22
∆Leptin 30 wks	*r* *P* n			1.000 41	-0.009 0.954 41	-0.211 0.196 39	0.051 0.765 36	-0.058 0.730 38	0.199 0.374 22	-0.091 0.687 22
∆Resistin 30 wks	*r* *P* n				1.000 41	0.048 0.770 39	-0.186 0.277 36	-0.143 0.393 38	0.103 0.647 22	-0.278 0.210 22
∆Free IGF-1 30 wks	*r* *P* n					1.000 40	0.103 0.556 35	0.178 0.299 36	0.356 0.104 22	-0.563 0.006 22
∆Total IGF-1 30 wks	r P n						1.000 40	0.043 0.812 33	-0.115 0.639 19	-0.128 0.601 19
∆IGFB3 30 wks	*r* *P* n							1.000 38	-0.089 0.695 22	-0.242 0.277 22
∆NGF 30 wks	*r* *P* n								1.000 22	-0.215 0.336 22

Correlation after 30 weeks between change (∆) in fatigue (∆FIQ and ∆MFIGF) and change in serum free IGF-1, serum levels of IGFB3, adipokines and neuropeptides. *r*, Spearman's correlation coefficient; *P*, *P*-value; n, number; FIQ, Fibromyalgia Impact Questionnaire; MFIGF, Multidimensional Fatigue Inventory subscale of General Fatigue; IGF, insulin-like growth factor; IGFB3, insulin-like growth factor-binding protein-3; NGF, nerve growth factor; NPY, neuropeptide Y.

**Table 7:** Change in fatigue in lean, overweight and obese patients versus change in adipokines, IGF-1 and neuropeptides

Lean patients		∆MFIGF 30 wks	∆Adiponectin 30 wks	∆Leptin 30 wks	∆Resistin 30 wks	∆Free IGF-1 30 wks	∆Tot IGF-1 30 wks	∆IGFB3 30 wks	∆NPY 30 wks	∆NGF 30 wks
∆FIQ Fatigue 30 weeks	*r* *P* n	0.268 0.521 8	0.541 0.210 7	-0.432 0.333 7	-0.685 0.090 7	0.145 0.784 6	0.812 0.05 6	0.086 0.872 6	1.000 2	1.000 2
∆MFIGF 30 weeks	*r* *P* n	1.000 8	0.275 0.550 7	-0.220 0.635 7	-0.239 0.606 7	0.059 0.912 6	0.706 0.117 6	0.177 0.738 6	1.000 2	1.000 2

**Overweight patients**		**∆MFIGF** **30 wks**	**∆Adiponectin** **30 wks**	**∆Leptin** **30 wks**	**∆Resistin** **30 wks**	**∆Free IGF-1** **30 wks**	**∆Tot IGF-1** **30 wks**	**∆IGFB3** **30 wks**	**∆NPY** **30 wks**	**∆NGF** **30 wks**

∆FIQ Fatigue 30 wks	*r* *P* n	0.530 0.008 24	-0.219 0.315 23	-0.099 0.654 23	0.270 0.213 23	-0.021 0.924 22	0.213 0.380 19	0.260 0.232 23	0.394 0.164 14	-0.475 0.086 14
∆MFIGF 30 wks	*r* *P* n	1.000 24	-0.089 0.685 23	0.127 0.562 23	-0.265 0.222 23	-0.233 0.297 22	-0.012 0.962 19	0.104 0.635 23	0.384 0.176 14	-0.047 0.874 14

**Obese patients**		**∆MFIGF** **30 wks**	**∆Adiponectin** **30 wks**	**∆Leptin** **30 wks**	**∆Resistin** **30 wks**	**∆Free IGF-1** **30 wks**	**∆Tot IGF-1** **30 wks**	**∆IGFB3** **30 wks**	**∆NPY** **30 wks**	**∆NGF** **30 wks**

∆FIQ Fatigue 30 wks	*r* *P* n	0.283 0.373 12	-0.753 0.019 9	0.084 0.831 9	-0.326 0.391 9	-0.711 **0.032** 9	-0.261 0.498 9	0.072 0.878 7	0.154 0.805 5	-0.205 0.741 5
∆MFIGF 30 wks	*r* *P* n	1.000 12	-0.210 0.587 9	0.647 0.060 9	-0.815 0.007 9	-0.025 0.949 9	-0.418 0.263 9	0.786 0.036 7	-0.900 **0.037** 5	0.500 0.391 5

Correlation after 30 weeks between change (∆) in fatigue (∆FIQ and ∆MFIGF) and change in serum free IGF-1, serum levels of IGFB3, adipokines and neuropeptides in lean patients, overweight and obese patients. *r*, Spearman's correlation coefficient; *P*, *P*-value; n, number; FIQ, Fibromyalgia Impact Questionnaire; MFIGF, Multidimensional Fatigue Inventory subscale of General Fatigue; IGF, insulin-like growth factor; IGFB3, insulin-like growth factor-binding protein-3; NGF, nerve growth factor; NPY, neuropeptide Y.

### Type 1 error

Analyses of baseline data, changes in fatigue, levels of adipokines and IGF levels (Tables 1, 2, 3, and text) comprised a total of 121 comparisons and the upper level of the number of false significant results was 5.10, which means that five of the significant results might be false.

Correlations at baseline (Tables 4 and 5, and text), comprised a total of 57 comparisons and the upper level of the number of false significant results was 2.26, which means that two significant results might be false. Correlations with regard to change (Tables 6 and 7, and text), comprised a total of 93 comparisons and the upper level of number of false significant results was 4.21, which means that four significant results might be false.

## Discussion

Fatigue is a debilitating and common health problem in FM and in many autoimmune rheumatic diseases, influencing quality of life, work ability and motivation to exercise. The cause of fatigue is multifactorial and poorly understood. Suggested causes of chronic fatigue include central and peripheral neuropeptides and cytokines, endocrine dysregulation and secondary effects due to pain, depression and sleep disturbance[3,18,55,56,67].

Aerobic exercise, together with pharmacological treatment, is one of the cornerstones of treatment for FM [68], and many patients with FM report lower levels of fatigue after a lengthy exercise period [63,69]. In this group of women with FM, the response to aerobic exercise on fatigue was related to levels of BMI. Lean patients already reported significantly reduced fatigue after 15 weeks of exercise. The response to exercise in overweight and obese patients was delayed, but a significant reduction in fatigue was found after six months. An association between BMI and fatigue in FM has previously been reported [70], and a high BMI together with inactivity also increases the risk for development of FM [71]. In our material, the overweight group reported lower levels of fatigue than the lean group. Fatigue levels between the lean and obese groups did not significantly differ.

We used two different instruments to rate fatigue [72]. Both ratings of general fatigue reflect symptom severity, but somewhat different aspects. The FIQ rates the global feeling of fatigue, possibly including a feeling of pain, and the MFIGF estimates fatigue in relation to feeling fit, tired and rested.

We found evidence of a positive role for total and free bioactive IGF-1 on fatigue. This is in line with previous reports that IGF-1 has a protective role in FM [17-19,73] promoting adaptation and neuroplasticity in the central nervous system [20,21]. Baseline levels of resistin in CSF were negatively correlated with fatigue. The same pattern, although not significant, was seen for resistin in serum. Increased resistin after 6 months correlated with reduced fatigue. Thus, the increase in resistin during exercise appears to improve fatigue, and the positive effects may be especially important in obese patients. Resistin represents a potential link between inflammation and metabolism and can stimulate TLR4 [36] as well as promote IGF-1 receptor signaling [37]. To the best of our knowledge, resistin has not previously been studied in relation to fatigue.

We also found evidence of a role for adiponectin, leptin and NPY in the reduced fatigue after exercise. Serum leptin and cerebrospinal adiponectin were both associated with low fatigue at baseline, and change in adiponectin correlated with reduced fatigue. NPY correlated with increased fatigue. Serum leptin is taken up via the blood-brain barrier and is a central regulator of energy levels with behavioral effects [43,42,48]. The arcuate nucleus of the hypothalamus is believed to be important in mediating these effects [43,74]. Different peripheral energy signals such as leptin and insulin [74] were found to activate different but overlapping subpopulations of arcuate NPY neurons. In line with this, the IGF-1-receptor is expressed in arcuate neurons and glial cells [75], and IGF-1 receptor activation is important for neuroplasticity in the arcuate hypothalamus [76]. Similarly, resistin can activate hypothalamic neurons and induce NPY expression in the hypothalamus [77]. Based on our findings, the roles of leptin and NPY in fatigue and the long-term effects of exercise merit further study.

This is an exploratory longitudinal study. Since we aimed to investigate the interaction of IGF-1 and adipokines in relation to BMI, the study includes many analyses. Due to multiple analyses, the significance level should be interpreted with caution, and the upper limit of the expected number of false sigificant results is presented in the Results section.

## Conclusions

Aerobic exercise reduced fatigue in all FM patients; this effect was achieved early in lean patients. In overweight and obese patients the reduction of fatigue was most pronounced after 6 months. Fatigue in FM patients is inversely correlated to resistin in serum and CSF, indicating a beneficial role of resistin. The long-term reduction of fatigue following exercise correlated with increased levels of resistin. The inverse correlation of resistin with reduced fatigue was more pronounced in obese FM patients. Changes in IGF-1 indicate a similar beneficial role on fatigue in obese patients. The results also indicate the involvement of leptin, adiponectin and NPY, although it is not clear how these signals may interact with each other in chronic fatigue.

## Abbreviations

ACR: American College of Rheumatology; BMI: body mass index; CNS: central nervous system; CSF: cerebrospinal fluid; ELISA: enzyme-linked immunosorbent assay; FM: fibromyalgia; FIQ: Fibromyalgia Impact Questionnaire; IL: interleukin; IGF-1: insulin-like growth factor-1; IGFBP3: insulin-like growth factor-binding protein-3; L3/L4: lumbar vertebrae 3 to 4; LIW: low-intensity walking; MFI-20: Multidimensional Fatigue Inventory; MFIGF: Multidimensional Fatigue Inventory subscale of General Fatigue; 6MWT: 6-minute walking test; NGF: nerve growth factor; NPY: neuropeptide Y; NW: Nordic walking; SP: substance P; TLR: toll-like receptor.

## Competing interests

The authors declare that they have no competing interests.

## Authors' contributions

JB: study conception and design, analysis and interpretation of data. ME: analysis and interpretation of data. MBo: acquisition, analysis and interpretation of data. KM: study conception, study design, acquisition, analysis and interpretation of data. All the authors were involved in the drafting of the article and revising it critically for important intellectual content. All the authors approved the final version of the article.
